# Supplementary material for: Artificial Intelligence for Early Detection of Chest Nodules in X-ray Images
Source: Biomedicines. 2022 Nov 7;10(11):2839. doi: 10.3390/biomedicines10112839 (PMC9687210; doi:10.3390/biomedicines10112839)
Supplement: Supplementary file 1 [file biomedicines-10-02839-s001.zip › biomedicines-1970072-supplementary.pdf]

Table S1. Relationship between nodule size and the probability of early detection.

	**Not early detected**	**Early detected by AI only**	**Early detected by radiologists only**	**Early detected by both AI and radiologists**
Nodule size, N(% of same size nodules)				
6-10mm	4 (25)	4 (25)	7 (43.7)	1 (7.3)
10-20mm	44 (29.5)	33 (22.1)	29 (19.5)	43 (28.9)
20-30mm	24 (20.8)	26 (22.6)	21 (18.3)	44 (38.3)
>30mm	17 (16.5)	28 (27.2)	11 (10.7)	47 (45.6)
Total, N(%)	89 (23.2)	91 (23.8)	68 (17.8)	135 (35.2)

The probility of early detection increased while the nodule size increased. The AI model (ensemble model) performed better than radiologists in nodules >10 mm. The radiologists perfomred better in nodules <10 mm.

Table S2. Relationship between nodule size and the misclassification.

	Misclassified by AI	Misclassified by radiologist	Nodules for classification, N
Nodule size, N(% of same size nodules)			
6-10mm	11 (68.8)	8 (50)	16
10-20mm	73 (49.0)	77 (51.7)	149
20-30mm	45 (39.1)	50 (43.5)	115
>30mm	28 (27.2)	45 (43.7)	103
Total, N(%)	157 (41.0)	180 (47.0)	383

Both the AI model (ensemble model) and the radiologists perfomed better when nodule size increased.
